# Macrophage Senescence: A New Axis in Inflammaging and MASLD with Implications for Senolytic Therapy

**DOI:** 10.1093/geroni/igaf122.818

**Published:** 2025-12-31

**Authors:** Anthony Covarubbias

**Affiliations:** University of California, Los Angeles, Los Angeles, California, United States

## Abstract

DNA damage or other cellular stress can lead to an irreversible cell cycle arrest, known as cellular senescence in immune cells. While cellular senescence in immune cells can be beneficial in some contexts such as wound healing and development, it is now recognized as a major source of sterile inflammation in aging tissues via the secretion of inflammatory factors known as the senescence-associated secretory phenotype (SASP). Targeting senescent immune cells is therefore an emerging strategy for treating age-related diseases, however, the identity and function of specific senescent immune cell types remain unclear. Here, we identify p21^⁴^senescent macrophages as a key source of chronic inflammation in aging and in metabolic dysfunction-associated steatotic liver disease (MASLD). To further investigate macrophage senescence, we developed an in vitro model of DNA damage-induced macrophage senescence in primary mouse and human macrophages. Using a multi-omic approach, we leveraged these biomarkers to distinguish senescent macrophages from other established polarized states. We also found that DNA damage or metabolic stress induces stable macrophage senescence marked by an elevated SASP, mitochondrial dysfunction, and interferon signaling. These senescent macrophages accumulate in aged mouse livers, particularly in Kupffer cells, and are enriched in human cirrhotic liver tissue. Using transcriptomic profiling we identified a unique transcriptomic signature to identify macrophage senescence both in vitro and in vivo for both mice and humans. We also showed that senescent macrophages can be selectively targeted for depletion using senolytics, which reduces liver steatosis and inflammation in MASLD models, highlighting macrophage senescence as a major source of inflammaging and promising therapeutic target in age-related diseases.

